# Lower selfing rates in metallicolous populations than in non-metallicolous populations of the pseudometallophyte *Noccaea caerulescens* (Brassicaceae) in Southern France

**DOI:** 10.1093/aob/mcv191

**Published:** 2016-01-15

**Authors:** Mathilde Mousset, Patrice David, Christophe Petit, Juliette Pouzadoux, Clémence Hatt, Élodie Flaven, Ophélie Ronce, Agnès Mignot

**Affiliations:** ^1^Institut des Sciences de l’Évolution, Université de Montpellier, CNRS, IRD, EPHE CC 065, Place Eugène Bataillon, 34095 Montpellier cedex 05, France and; ^2^Centre d’Écologie Fonctionnelle et Évolutive, CEFE-UMR 5175, Campus CNRS, 1919 Route de Mende, 34293 Montpellier cedex, France

**Keywords:** Mating system, metallicolous, microsatellite markers, MLTR, *Noccaea caerulescens* (formerly *Thlaspi caerulescens*), pseudometallophyte, RMES, selfing, self-fertilization

## Abstract

**Background and Aims** The pseudometallophyte *Noccaea caerulescens* is an excellent model to study evolutionary processes, as it grows both on normal and on heavy-metal-rich, toxic soils. The evolution and demography of populations are critically impacted by mating system and, yet, information about the *N. caerulescens* mating system is limited.

**Methods** Mean selfing rates were assessed using microsatellite loci and a robust estimation method (RMES) in five metallicolous and five non-metallicolous populations of *N. caerulescens* in Southern France, and this measure was replicated for two successive reproductive seasons. As a part of the study, the patterns of gene flow among populations were analysed. The mating system was then characterized at a fine spatial scale in three populations using the MLTR method on progeny arrays.

**Key Results** The results confirm that *N. caerulescens* has a mixed mating system, with selfing rates ranging from 0·2 to 0·5. Selfing rates did not vary much among populations within ecotypes, but were lower in the metallicolous than in the non-metallicolous ecotype, in both seasons. Effective population size was also lower in non-metallicolous populations. Biparental inbreeding was null to moderate. Differentiation among populations was generally high, but neither ecotype nor isolation by distance explained it.

**Conclusions** The consequences of higher selfing rates on adaptation are expected to be weak to moderate in non-metallicolous populations and they are expected to suffer less from inbreeding depression, compared to metallicolous populations.

## INTRODUCTION

Organisms with the ability to grow on extremely toxic soils, such as mine wastes or mine smelters, have triggered a lot of interest recently (McNeilly and Antonovics, 1968; Antonovics *et al*., 1971; Bradshaw, 1984; Macnair, 1987; Pauwels *et al*., 2008). *Noccaea caerulescens* is one such organism, and is now a model species for unravelling the genetic and physiological mechanisms of metal tolerance and hyperaccumulation (Assunção *et al.*, 2003*b*; Hanikenne and Nouet, 2011, and references therein), with potential applications in phytostabilization or phytoextraction (Chaney *et al.*, 1997; but see Ernst, 2005). *Noccaea caerulescens* is a pseudometallophyte herb, as it can grow on both metal-rich soils and other types of soils. It is constitutively tolerant to soils containing high concentrations of trace elements such as cadmium (Cd), zinc (Zn) and lead (Pb) (e.g. Meerts and Van Isacker, 1997; Escarré *et al.*, 2000) and is also an hyperaccumulator, because it can accumulate extremely large quantities of nickel (Ni), Zn and Cd in its tissues (Assunção *et al.*, 2003*a*; Verbruggen *et al.*, 2009, and references therein). It is related to *Arabidopsis thaliana*, which conveniently makes some genetic resource transfers possible (Rigola *et al.*, 2006). *Noccaea caerulescens* is also an excellent model to investigate eco-evolutionary processes such as the adaptation to stressful environments or the evolution of local adaptation (Dubois *et al.*, 2003; Dechamps *et al.*, 2007; Jiménez-Ambriz *et al.*, 2007; Noret *et al.*, 2007; Besnard *et al.*, 2009; Fones *et al.*, 2013).

Some populations of *N. caerulescens* have higher levels of tolerance to trace elements than others (Meerts and Van Isacker, 1997; Escarré *et al.*, 2000; Assunção *et al.*, 2003*a*), leading to the description of different ecotypes based on edaphic conditions: in particular, a metallicolous (or calamine) ecotype that grows on metalliferous soils such as former mining sites, more tolerant but accumulating less Zn, and a non-metallicolous ecotype that grows on non-metalliferous soils, less tolerant but accumulating more Zn (Meerts and Van Isacker, 1997; Escarré *et al.*, 2000; Assunção *et al.*, 2003*a*). Previous studies showed genetic differentiation between ecotypes at some candidate loci involved in metal metabolism (Besnard *et al.*, 2009), as well as local adaptation of metallicolous populations, based on both reciprocal transplants in natural populations (Dechamps *et al.*, 2008) and common garden experiments (Meerts and Van Isacker, 1997; Dechamps *et al.*, 2007; Jiménez-Ambriz *et al.*, 2007). However, these ecotypes could not be distinguished using neutral markers (Koch *et al.*, 1998; Dubois *et al.*, 2003; Jiménez-Ambriz *et al.*, 2007), suggesting either independent recurrent adaptation to different edaphic conditions or enough gene flow between ecotypes to homogenize genomes except around genes involved in local adaptation.

The mating system, especially the selfing rate, is a key factor to consider in this context. Indeed, it affects both the ecology and evolution of organisms, and in particular their adaptation to extreme conditions in heterogeneous environments (Levin, 2010), such as metalliferous and non-metalliferous soils on which the self-compatible species *N. caerulescens* grows. For example, autonomous selfing provides reproductive insurance when pollinators or mates are scarce, which could help in colonizing new habitats (Baker, 1955; Levin, 2010, Pannell, 2015; but see Cheptou, 2012). This positive demographic effect may be counterbalanced by inbreeding depression, i.e. the lower fitness of selfed compared with outcrossed progeny, which may increase the demographic vulnerability of small and inbred populations. Note, however, that if inbreeding depression affects mostly the early stages of the life cycle in populations where the number of juveniles greatly exceeds the carrying capacity, it may have very little impact on the prospects of population survival.

Selfing affects all evolutionary forces by modifying patterns of gene flow, decreasing recombination efficacy, increasing genetic drift and exposing genetic variation to selection. This may have both positive and negative effects on population survival and adaptation. On the one hand, recessive mutations in inbred individuals can be purged through selection when deleterious, and be fixed when beneficial (Caballero and Hill, 1992). In the latter case, this would speed up adaptation to new environments (Glémin and Ronfort, 2013). On the other hand, selfing reduces effective population size (Nordborg, 2000) and effective recombination (Holden 1979, Hartfield and Glémin, 2014), and consequently may lead to a loss of standing genetic diversity, and compromise the adaptive capacities of highly selfing species in the long term (Stebbins, 1957; Wright *et al.*, 2013; Lande and Porcher, 2015). Finally, the mating system may constrain local adaptation in heterogeneous environments by affecting gene flow across habitats with divergent selection (Lopez *et al.*, 2008), such as contaminated soils next to non-contaminated soils. In the classic study on the joint evolution of mating system and local adaptation, Antonovics (1968) found that *Agrostis tenuis* and *Anthoxanthum odoratum* plants growing in a mine had higher autonomous selfing rates than did plants growing on a nearby non-contaminated pasture, consistent with his theoretical prediction of an advantage to selfing in such heterogeneous habitats. However, further theoretical work showed that the relationships between local adaptation and selfing rates could be complicated by the joint evolution of inbreeding depression in heterogeneous environments (Epinat and Lenormand, 2009; Ronce *et al.*, 2009). In particular, Epinat and Lenormand (2009) predicted that the evolution of increased selfing in heterogeneous habitats may be impeded by the evolution of higher inbreeding depression at the local adaptation locus. In addition, after the initial results of Antonovics (1968), replicated by Cuguen *et al.* (1989) and Lefèbvre (1970) (but see Lefèbvre, 1973, 1976; Dulya and Mikryukov, 2015), the accumulated empirical evidence did not provide overall support to the idea of a general and strong association between selfing and plant local adaptation (for two meta-analyses, see Leimu and Fischer, 2008; Hereford, 2010).

Despite its key role in the ecology and adaptation to heterogeneous habitats, the mating system of *N. caerulescens* has not been characterized in detail. The species is self-compatible, and, based on *F*_IS_ values, has a mixed mating system (Koch *et al.*, 1998; Dubois *et al.*, 2003; Jiménez-Ambriz *et al.*, 2007; Besnard *et al.*, 2009). Dubois *et al*. (2003) found that, in contrast to the finding of Antonovics (1968), non-metallicolous populations had higher inbreeding than metallicolous populations. This result was, however, derived from estimations of *F*_IS_ based on four enzymatic markers with limited polymorphism, and on the measurement of pollen–ovule ratios. Estimates of selfing rates based on fixation indexes suffer from several methodological biases (e.g. in the presence of null alleles), and are sensitive to population characteristics such as sub-structuration (Jarne and David, 2008), biparental inbreeding in particular (Jarne and David, 2008; Wang *et al.*, 2012). Selfing rates in plants with a mixed mating system are known to vary from one season to another (e.g. Cheliak *et al*., 1985; Barrett *et al*., 1993; Eckert *et al*., 2009; Coates *et al*., 2013), due to environment variation affecting both pollinator and plant communities. Traits potentially affecting the mating system such as the height of flowering stalks vary significantly across years in natural populations of *N. caerulescens* (Jiménez-Ambriz *et al*., 2007). Estimating mating system parameters in more than one reproductive season would thus allow a more robust evaluation of the variation of mating system between populations and of the stability of these patterns through time.

Here we present a detailed analysis of the mating system in metallicolous and non-metallicolous populations of *N. caerulescens*, focusing on a set of geographically close populations to minimize potential causes of variation in the mating system. Ten populations from the Causses and Cévennes region in Southern France were investigated using 14 microsatellite markers. The ten populations were used for two consecutive reproductive seasons, and one population was used for four seasons. We described patterns of gene flow among populations, as reflected by their genetic structure, and estimated their effective size. Selfing rates within each population were estimated using a method robust to scoring artefacts, and based on correlations in homozygosity across loci (RMES; David *et al.*, 2007). To gain more insight into the relative role of selfing and other forms of inbreeding, we further analysed a distinct data set, corresponding to progeny arrays of genotypes in three of the ten populations, using the MLTR method (Ritland, 2002).

## MATERIALS AND METHODS

### Species presentation

*Noccaea caerulescens* (J. Presl & C. Presl F.K. Mey) is a pseudometallophyte from the *Brassicaceae* family. It is annual to perennial, flowers in spring and grows one to several dozen inflorescences with small white to light purple flowers. Its main pollinators in the studied area have not yet been extensively described, but observations in several populations for several years reveal that Hymenoptera (domesticated and wild bees, bumble-bees), small Diptera and Lepidoptera visit *N. caerulescens* flowers (I. Decombeix, A. Mignot, C. Petit, L. Prat, ISEM, Montpellier, pers. comm.). These pollinators broadly correspond to previous observations of hover flies, flies, Lepidoptera and bees visiting flowers in Germany and Great Britain (Knuth, 1908; Rileys, 1956). Most individuals from populations in Southern France exhibit an annual life cycle (Dubois, 2005): seeds produced during a given spring and summer will germinate the following autumn after the first abundant rains and will spend the winter as rosettes; about 30 % of the rosettes will survive and flower the following spring and about 11 % will survive and stay in the non-reproductive state the following spring (Dubois, 2005). In the studied area, situated north of Montpellier, *N. caerulescens* grows on relatively open substrate. Metallicolous populations occur on nutrient-poor, schistous or dolomitic soils, while non-metallicolous populations grow on limestone soils.

### Methods to estimate selfing rates

Different approaches are available to study mating systems, using different types of data and different assumptions (Jarne and David, 2008). We used two of them to better characterize the mating system of *N. caerulescens*. The RMES software (David *et al.*, 2007), which is based on a population structure approach, infers the mean population selfing rate using grown individuals sampled directly in the populations. Its estimation integrates selfing rates across several generations. The MLTR software (Ritland, 2002), which is based on a progeny array approach, uses family structured data to infer population mating system parameters from seeds collected in the populations. In contrast to the former approach, its estimated selfing rate represents a snapshot of the mating structure in the previous generation, and is usually less affected by early acting inbreeding depression because progeny are typically sampled as seedlings.

### Sampling for the population structure approach

**Fig. 1.**
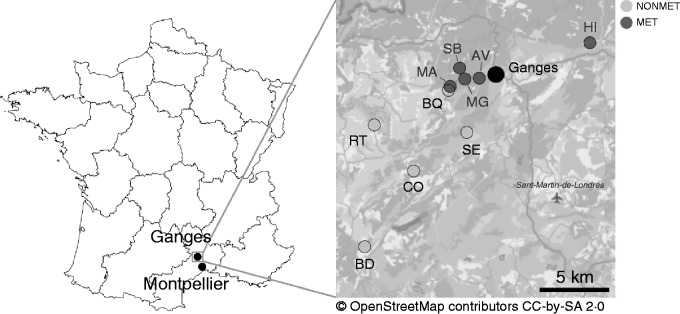
Geographical location of the ten natural populations of *Noccaea caerulescens* sampled in the South of France in 2012 and 2013. Non-metallicolous populations are represented in light grey, and metallicolous populations in dark grey. Background maps: © OpenStreetMaps Contributors. AV, Avinières; BD, Saint Baudille; BQ, Baraquette; CO, Coulet; HI, Saint Hippolyte; MA, Malines; MG, Moyen-Âge; RT, Navacelles Route; SB, Saint Bresson; SE, Séranne.

We chose five populations with high heavy metal concentrations (hereafter called metallicolous populations or MET) and five populations on calcareous soil (non-metallicolous populations or NONMET) in the Causses and Cévennes, the southern part of Massif Central and north of Montpellier (France). All sites (Fig. 1; Table 1) were close to Ganges and separated by <1–38 km. We sampled leaves on plants in May–June 2013, and in February–April 2014. These plants were at the rosette or flowering stage and were assumed to represent mostly the offspring of the 2012 and 2013 reproductive seasons, respectively. Plants were sampled in approximately the same areas in the populations for the two years. For all sites, leaves were collected on 40–50 plants along a random walk, on individuals separated by >1 m whenever possible to limit sampling of related individuals; they were subsequently oven dried (40 °C during 48–72 h), and kept in silica gel. In the Saint Bresson (SB) population, we sampled approx. 50 plants each year twice. As the sample size affects the estimation of genetic diversity, we provide separate estimates for each sample (their size is similar to that of the other populations), but we pool the two samples to estimate selfing rates with more precision. For this population, we also sampled leaves during spring 2011 and 2012 and were thus able to obtain selfing rate estimates for four consecutive reproductive seasons.

**Table 1.** Name, location and sample size of the sampled natural populations of *Noccaea caerulescens*

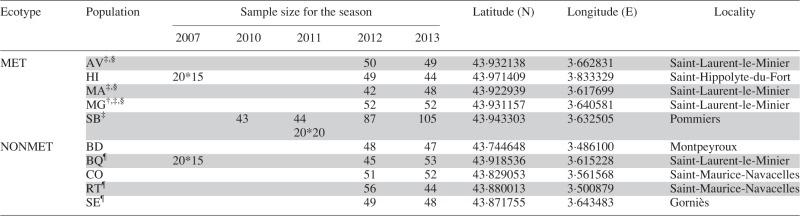

Years represent the reproductive season studied. Samples correspond to leaves, except for the progeny array approach for which seeds have been collected. For these samples, numbers separated by an asterisk represent the number of families and the number of offspring genotyped per family. Concentrations of trace elements can be found in the publications listed below, when available.

MET, metallicolous; NONMET, non-metallicolous. AV, Avinières; BD, Saint Baudille; BQ, Baraquette; CO, Coulet; HI, Saint Hippolyte; MA, Malines; MG, Moyen-Âge; RT, Navacelles Route; SB, Saint Bresson; SE, Séranne.

^†^Also known as Petra Alba in Escarré *et al.* (2011).

^‡^Dubois *et al.* (2003).

^§^Escarré *et al*. (2011).

^¶^Dubois (2005).

### Sampling for the progeny array approach

Thirteen to twenty seeds from several inflorescences of the same plant were collected at maturity (June–July) on 20 mothers per population in Saint Hippolyte (HI; MET) and Baraquette (BQ; NONMET) in 2007, and in SB (MET) in 2011. Seeds from each family were sown in the year of harvest, on burnt clay (HI and BQ) or vermiculite (SB) in randomized containers in a temperate glasshouse at the experimental garden of LabEx CeMEB (Plateforme des Terrains d’Expériences du LabEx CeMEB). Seedlings were collected at the four-leaf stage, oven dried and genotyped. The mother genotype was available only for SB; it was thus inferred for the other populations.

### Microsatellite genotyping

Neutral diversity was assessed using 15 microsatellite loci organized in two multiplexes (NcM1 and NcM2) and following the protocol described in Mousset *et al*. (2015). Two microsatellite markers, 6g4 and 6e4 (Jiménez-Ambriz *et al.*, 2007), were added to NcM1 and NcM2, respectively, following the same protocol (6g4, fluorochrome PET, final concentration 0·8 μm; 6e4, fluorochrome 6FAM, final concentration 0·4 μm). DNA was extracted using a classic cetyltrimethylammonium bromide (CTAB) protocol (Doyle and Doyle, 1990), amplified using PCR and genotyped using capillary electrophoresis on an ABI PRISM 3130 xl Genetic Analyzer (SB 2010, 2011 and progeny array 2011) and an ABI PRISM 3500 xl Genetic Analyzer (all other populations). Raw data were analysed using GeneMapper® version 5.0 (Applied Biosystems). The use of a different Genetic Analyzer led to different migration conditions, and thus different bins. As a consequence, allelic frequencies from SB 2010 and 2011, and progeny arrays from 2011 samples were not compared with other populations genotyped later. Nevertheless, the RMES method relies only on the presence or absence of heterozygotes within populations to compute mean selfing rates. We were thus able to compare the 2010 and 2011 estimates of mean selfing rates with the 2012 and 2013 estimates using the population structure approach.

In the 2011 SB population, null alleles were obvious for some markers in some families; Ncpm31 and Tc-up2 were removed from the analysis, and thus the analysis was performed on 13 loci. Due to amplification issues, 6g4 was barely readable in the genotypes in the ten populations of 2012 and 2013 and thus were removed from their analyses (which was thus performed on 14 loci).

### Genetic diversity

We used GENETIX (version 4.05, Belkhir *et al.*, 2004) to compute allelic frequencies, number of alleles, inbreeding coefficient *F*_IS_ [using the Weir and Cockerham (1984) method with 1000 bootstrap 95 % confidence intervals (CIs)] and the amount of linkage disequilibrium between loci, and Genepop (Rousset, 2008) to test for linkage equilibrium among loci. Combining the information of these two software programs, no pair of loci had a statistically significant correlation coefficient higher than 50 % in more than two populations; therefore, we kept all loci in the following analyses. We used the permutation test in FSTAT (Goudet, 2005) to compare the observed and expected heterozygosity, *F*_IS_ and *F*_ST_, of the metallicolous and non-metallicolous ecotypes (5000 permutations of populations between ecotypes).

### Analysis of gene flow among populations

#### Partitioning of genetic variance.

We used Genepop to calculate pairwise *F*_ST_ values between pairs of populations (for each year) and the HIERFSTAT R package (Goudet, 2005; R Core Team, 2014) to partition genetic variance among ecotypes, populations and plants (95 % CIs were based on 1000 bootstraps).

#### Isolation by distance.

We tested for the presence of isolation by distance among populations using the R package ‘fields’ (Nychka *et al.*, 2014) to calculate the geographic distance matrix among populations based on their GPS coordinates. We then tested for the presence of isolation by distance each year with Mantel tests in the ‘adegenet’ R package (Jombart and Ahmed, 2011), within each ecotype and on the whole data set. Additionally, following the guidelines provided by Rousset (1997), we regressed *F*_ST_/(1 – *F*_ST_) on the geographic distance.

#### Principal component analysis.

To better describe the structure of genetic diversity, we calculated a centred, non-scaled principal component analysis (PCA) on allele frequencies in 2012 and 2013 using the R packages ‘adegenet’ and ‘ade4’ (Dray and Dufour, 2007; Jombart and Ahmed, 2011).

### Mating system analysis

#### Population structure approach (RMES).

We estimated mean population selfing rates with the RMES software (Robust Multilocus Estimate of Selfing, version 2009; David *et al.*, 2007). RMES uses the distribution of heterozygosity across all loci to infer selfing rates, and therefore provides estimates that are robust to several common problems of microsatellite data, such as the presence of null alleles or partial dominance. RMES maximizes likelihood (precision used: 0·0001) to estimate selfing rates (along with its 95 % CI) and computes the likelihood profile (i.e. the likelihood of all possible selfing rates at regularly spaced intervals). We used both pieces of information in two different approaches to compare selfing rates between populations, ecotypes and years of sampling.

Likelihood ratio tests (LRTs) can been used to test for differences in selfing rates between populations (a single selfing rate, *s*, for all populations vs. a different *s* for each population; David *et al*., 2007). Our problem was different as we wanted to test for differences in the distributions of *s* (and especially for differences in the mean of the distributions) between metallicolous and non-metallicolous populations, the ‘ecotype’ being equivalent to a fixed factor and the ‘population within ecotype’ being a source of random variation, similarly to a mixed model analysis of variance (ANOVA). To implement this approach we assumed that in each ecotype, population selfing rates (*s*) followed a logit-normal distribution and looked for values of the mean and standard deviation of logit(*s*) that maximize the likelihood over all data. We also maximized the likelihood under specific constraints, e.g. assuming that the two ecotypes had the same standard deviation and/or the same mean, or that they were equal to pre-defined values (e.g. 0). Models with and without a relevant constraint were then compared using standard LRTs. To fit these models, we used a Mathematica 9 routine (Wolfram Research, Inc., 2012) that computes the relevant likelihoods by combining population-specific likelihood profiles given by RMES and uses a simple iterative procedure to maximize them. Analyses were performed separately for the years 2012 and 2013. We found that the among-population standard deviation did not differ between ecotypes, and from zero in either ecotype. Therefore, we tested separately for the 2012 and 2013 data whether mean *s* were significantly different between ecotypes by performing LRT on models with a single or two means for the two ecotypes, assuming s.d. = 0 within each ecotype.

To investigate temporal variation in mating system across years, we tested separately for the two ecotypes if they had the same selfing rate in the two sampled reproductive seasons. Using the same framework as before, we used LRT to compare, for each ecotype separately, a model allowing a different *s* per season with a model estimating a single overall *s*. Finally, we similarly tested for variation by year in selfing rates in SB for the four seasons.

#### Progeny array approach.

We used MLTR (version 3.3; Ritland, 2002) on the progeny structured data sets to estimate mating system parameters using maximum likelihood estimations in HI, BQ and SB. We used the Newton–Raphson algorithm to obtain single locus and multilocus estimates of selfing rate (*s*_s_ and *s*_m_), correlations of selfing between families *r*_t_ (variance in selfing rates among mother plants.), correlations of paternity among siblings *r*_p_ (the inverse of the estimated number of fathers for the progeny of a single mother) and paternal inbreeding coefficient, *F*. We used 1000 bootstraps with whole families resampling to estimate 95 % CIs for these estimates. Comparing single locus- and multilocus-based selfing rates enables us to estimate the contribution of biparental inbreeding in the population, i.e. mating among related plants. Estimates of mating system parameters when assuming equal allele frequencies in the pollen cloud and ovule pool, and when relaxing this assumption, were similar. As the CIs were larger in the latter case, we used the most parsimonious model.

### Effective population size

We took advantage of the two sampling years to estimate variance effective size in each population (ten populations sampled for two consecutive reproductive seasons). We used the MLNe software (Wang and Whitlock 2003) to estimate effective size, with the maximum likelihood approach, assuming no immigration in populations. This method is based on the fact that, as effective size decreases, allele frequencies vary more widely through time due to drift effects.

## RESULTS

### Within-population genetic diversity

The mean number of alleles per locus was 5·4 ± 1 in metallicolous populations and 6·2 ± 0·7 in non-metallicolous populations (Table 2). Population allelic frequencies within each population were similar across the two sampled years. Pairwise *F*_ST_ within populations between the two sampling years was therefore small (<0·06). Mean genetic diversity was higher in non-metallicolous populations than in metallicolous populations, although the difference was not significant (MET 2012, 0·54 ± 0·10 and MET 2013, 0·55 ± 0·11; NONMET 2012, 0·63 ± 0·04 and NONMET 2013, 0·63 ± 0·04; permutation test, *P*_2012_ = 0·13, *P*_2013_ = 0·16; Table 2). Mean observed heterozygosity was similar in metallicolous and non-metallicolous populations (MET 2012, 0·43 ± 0·08 and MET 2013, 0·46 ± 0·10; NONMET 2012, 0·42 ± 0·07; NONMET 2013, 0·41 ± 0·06; permutation test, *P*_2012_ = 0·89, *P*_2013_ = 0·55; Table 2). *F*_IS_ was lower in metallicolous populations than in non-metallicolous populations (MET 2012, 0·22 ± 0·03 and MET 2013, 0·19 ± 0·04; NONMET 2012, 0·35 ± 0·11 and NONMET 2013, 0·37 ±0·07; *P*_2012_ = 0·04; *P*_2013_ = 0·007, Table 2). The mean inbreeding coefficient *F*_I__S_ was different from zero in all populations for the two years (except in the two SB sub-samples in 2012), indicating a general deficit in heterozygotes. This confirms the mixed mating system of *N. caerulescens* and suggests that metallicolous populations may have a less inbred mating system than do non-metallicolous populations.

**Table 2.** Sample size and genetic diversities in all ten populations of *Noccaea caerulescens* in 2012 and 2013

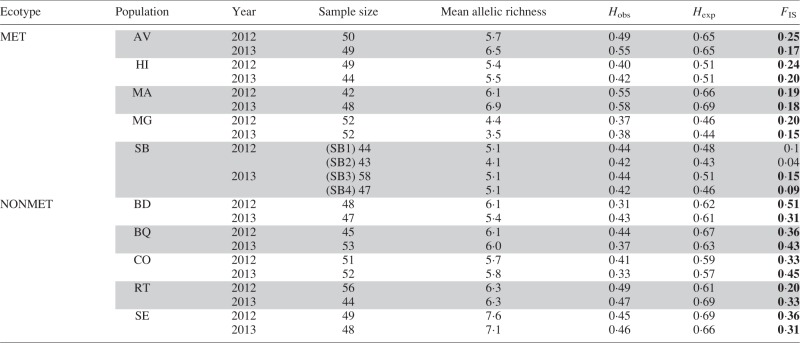

*F*_IS_ values in bold are statistically different from zero.

*H*_obs_, observed heterozygosity; *H*_exp_, expected heterozygosity; *F*_IS_, inbreeding coefficient. MET, metallicolous; NONMET, non-metallicolous. AV, Avinières; BD, Saint Baudille; BQ, Baraquette; CO, Coulet; HI, Saint Hippolyte; MA, Malines; MG, Moyen-Âge; RT, Navacelles Route; SB, Saint Bresson; SE, Séranne.

SB1, SB2, SB3 and SB4 correspond to different samplings in the SB population in the two seasons.

### Among-population genetic structure

Population differentiation was relatively high in general given the short geographical distances between them, but ecotype structuration did not account for it. Pairwise *F*_ST_ ranged from 0·07 to 0·42 in 2012 and 2013 (Supplementary Data Table S1). Mean pairwise *F*_ST_ was 0·29 (s.d. = 0·1) among metallicolous populations in both 2012 and 2013, and 0·14 (s.d. = 0·02) and 0·16 (s.d. = 0·03) among non-metallicolous populations in 2012 and 2013, respectively. The difference in *F*_ST_ was significant in 2012 (*P* = 0·02) but not in 2013 (*P* = 0·11). Hierarchical fixation indexes, as reported in Table 3 for both years, indicated differentiation among populations within ecotypes but very little structuration by ecotypes. The high within-population fixation index is consistent with the high *F*_IS_ measured in all populations.

**Table 3.** Hierarchical fixation indexes between ecotypes, populations and individuals of *Noccaea caerulescens* in 2012 and 2013

Source of variation	Year	Fixation index (CI)
Among ecotypes	2012	0·04 (0·01–0·06)
	2013	0·04 (0·01–0·07)
Among populations within ecotypes	2012	0·25 (0·2–0·29)
	2013	0·24 (0·20–0·28)
Among individuals among populations	2012	0·28 (0·26–0·3)
	2013	0·28 (0·25–0·32)

CI, confidence intervals (1000 bootstraps); *F*, hierarchical fixation index.

Isolation by distance could not be detected in the data set. Partial Mantel tests for each ecotype and for each year revealed no pattern of isolation by distance (2012 MET, *P* = 0·19; NONMET, *P* = 0·07; all populations *P* = 0·28; 2013 MET, *P* = 0·22; NONMET, *P* = 0·21; all populations *P* = 0·28). Similarly, the slopes of the regression of *F*_ST_/(1 – *F*_ST_) on the logarithm of distance were not significantly different from zero, within metallicolous populations, within non-metallicolous populations or among all populations (Supplementary Data Fig. S1), thus confirming the absence of isolation by distance, at least at the scale of the few kilometres separating our populations.

The PCA did not discriminate ecotypes (Supplementary Data Fig. S2); instead, the principal components each separated one of the metallicolous populations from all other populations, suggesting greater genetic originality of the metallicolous populations. The first four components explained only 27·4 % of the inertia in 2012 and 23·2 % in 2013. None of these axes discriminated all metallicolous populations from all non-metallicolous populations. Most genetic variation was thus not explained by ecotype differentiation, and each metallicolous population tended to be differentiated from all other populations.

### Effective population size

Estimated effective population size varied quite a lot between populations and was, on average, smaller in non-metallicolous populations than in metallicolous populations (Table 4). In one population, the effective population size was at the maximum the software can compute. Several upper values of CIs reach the upper limit of estimation, all in metallicolous populations. Taken together, these results suggest moderate to large variance effective size in metallicolous populations (>100) and small to moderate variance effective size for non-metallicolous populations, generally <100 and with size as small as 23.

**Table 4.** Variance in effective size in *Noccaea caerulescens* estimated by MLNe using the maximum likelihood approach, with 95 % confidence intervals

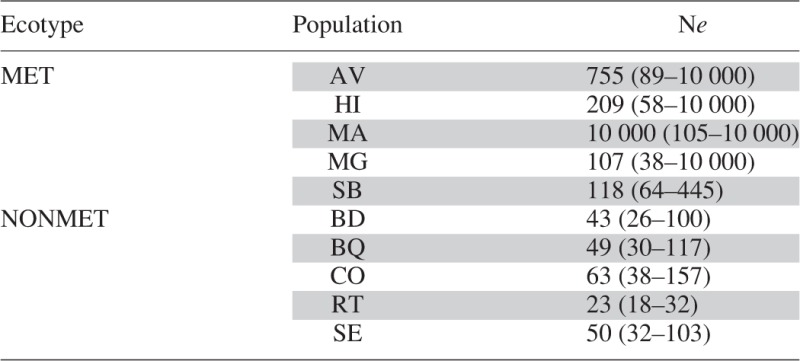

MET, metallicolous; NONMET, non-metallicolous. AV, Avinières; BD, Saint Baudille; BQ, Baraquette; CO, Coulet; HI, Saint Hippolyte; MA, Malines; MG, Moyen-Âge; RT, Navacelles Route; SB, Saint Bresson; SE, Séranne.

### Mating system

**Fig. 2.**
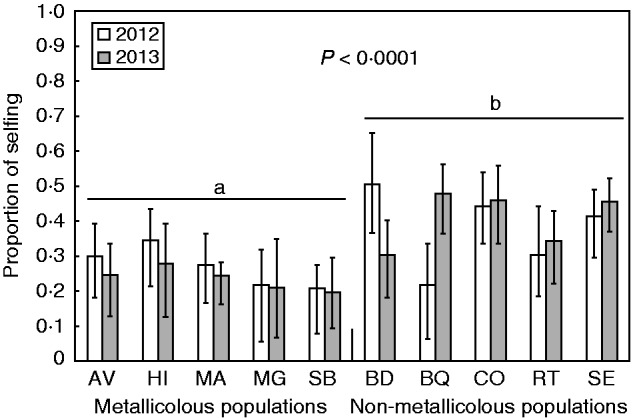
Mean selfing rate in the ten natural populations of *Noccaea caerulescens* sampled in the South of France in 2012 and 2013. Estimates were obtained using the maximum likelihood method in RMES. Error bars represent 95 % bootstrap confidence intervals. Selfing rates represented by different letters are statistically significantly different. Analysis was performed on 14 loci.

Populations of *N. caerulescens* in the studied area exhibited a clear mixed mating system: selfing rates estimated by RMES ranged between 0·2 and 0·5, depending on populations and years (Fig. 2), and the mean selfing rate of populations was 0·25 ± 0·05 in metallicolous populations and 0·39 ± 0·09 in non-metallicolous populations. Population selfing rates estimated by RMES were consistently lower than those generated via *F*_IS_ (Supplementary Data Fig. S3), and ecotype selfing rates were approx. 25 % smaller in RMES compared with *F*_IS_ based estimations, for both ecotypes.

We found clear differences between ecotypes. The LRTs showed that, in both 2012 and 2013, logit-normal distributions of selfing rates had different means for the two ecotypes, but equal standard deviations (Table 5). This standard deviation converged towards zero (due to numerical precision, estimated standard deviations cannot be smaller than 0·002) so we simplified the models by assuming s.d. = 0 for both ecotypes (see the Materials and Methods). Using RMES both in 2012 and in 2013, we found significant differences in selfing rates between the two ecotypes (Tables 6 and 7), as a model estimating two different selfing rates in the two ecotypes clearly outperformed a constrained model with equal selfing rates across ecotypes.

**Table 5.** Description of the models fitted on selfing rates in *Noccaea caerulescens* using the full information of the likelihood profiles, and result of model comparisons based on log likelihood tests

Year	Mean(s)	s.d.	Log likelihood	Difference in deviance	d.f.	*P*-value
2012	*μ*_MET-2012,_ *μ*_NONMET-2012_	*σ*_MET-2012,_ *σ*_NONMET-2012_	−4·90			
	*μ***_MET-2012,_** *μ***_NONMET-2012_**	***σ*_2012_**	−4·97	0·14	1	0·71
	*μ*_2012_	*σ*_2012_	−7·31	4·68	1	**0·03**
2013	*μ*_MET-2013,_ *μ*_NONMET-2013_	*σ*_MET-2013,_ *σ*_NONMET-2013_	−2·86			
	*μ***_MET-2013,_** *μ***_NONMET-2013_**	***σ*_2013_**	−2·86	0·00	1	1
	*μ*_2013_	*σ*_2013_	−8·70	11·67	1	**0·001**

MET, metallicolous; NONMET, non-metallicolous; *μ*, mean of the logit-normal distribution; *σ*, standard deviation of the logit-normal distribution of selfing rates among populations.

The best models are in bold.

**Table 6.** Description of the models fitted on selfing rates in *Noccaea caerulescens* and results of model comparisons

Populations	Year	Estimated selfing rate(s)	Log likelihood	Delta deviance	d.f.	*P*-value
All pop	2012	*s*_MET__-2012_, *s*_NONMET__-2012_	−3366·1	6·5	1	**0·01**
	2012	*s*_2012_	−3369·3			
All pop	2013	*s*_MET__-2013_, *s*_NONMET__-2013_	−3739·8	14·3	1	**0·0002**
	2013	*s*_2013_	−3746·9			
MET	2012–2013	*s*_MET-2012_, *s*_MET-2013_	−3912·8	0·1	1	0·75
	2012–2013	*s*_MET_	−3912·9			
NONMET	2012–2013	*s*_NONMET-2012_, *s*_NONMET-2013_	−3193·0	0·2	1	0·65
	2012–2013	*s*_NONMET_	−3193·2			
All pop	2012–2013	*s*_MET__,_ *s*_NONMET_	−7106·1	21·7	1	**<0·0001**
	2012–2013	*s*	−7116·9			
SB	2010–2011–2012–2013	*s*_SB-2010_, *s*_SB-2011_, *s*_SB-2012_, *s*_SB-2013_	−2014·8	2·4	3	0·5
	2010–2011–2012–2013	*s*_SB_	−2016·0			

The models were fitted using maximum log likelihood computed in RMES, and compared using log likelihood ratio tests.

MET, metallicolous; NONMET, non-metallicolous; pop, population; s represents the constrained or unconstrained selfing rate estimated for one population or a group of populations.

The best models are in bold, and the value of underlined estimated selfing rates are provided in Table 7.

**Table 7.** Mean selfing rates within ecotype of *Noccaea caerulescens* for each year and 95 % likelihood confidence intervals

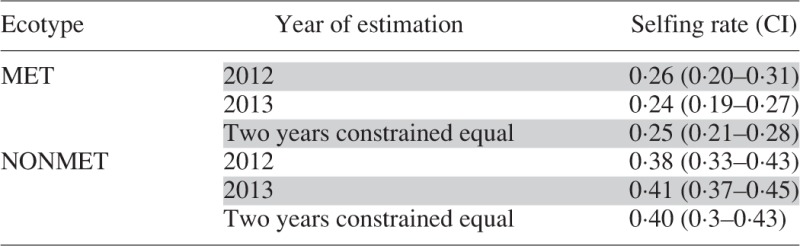

MET, metallicolous; NONMET, non-metallicolous.

**Fig. 3.**
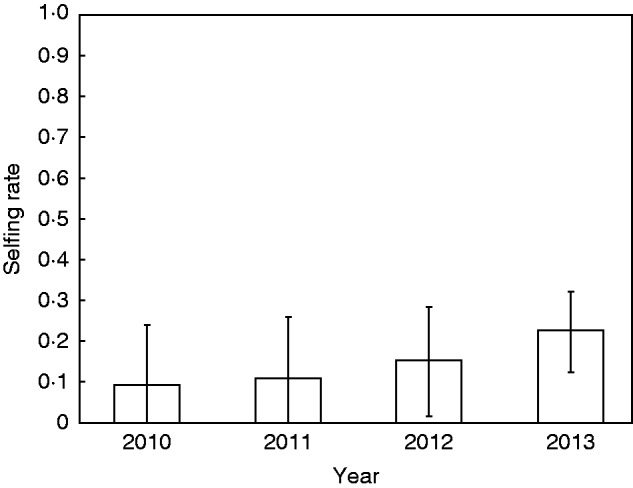
Mean selfing rate in the SB population of *Noccaea caerulescens*, for four consecutive seasons. Estimates were obtained using the maximum likelihood method in RMES. Error bars represent 95 % likelihood confidence intervals. Analysis was performed on 14 loci.

There was no evidence of temporal variation. Within each ecotype, we found no consistent significant difference in selfing rates across the two years in populations of the same ecotype (Tables 6 and 7). Similarly, no temporal variation was detected during four consecutive years in SB (Table 6; Fig. 3).

**Fig. 4.**
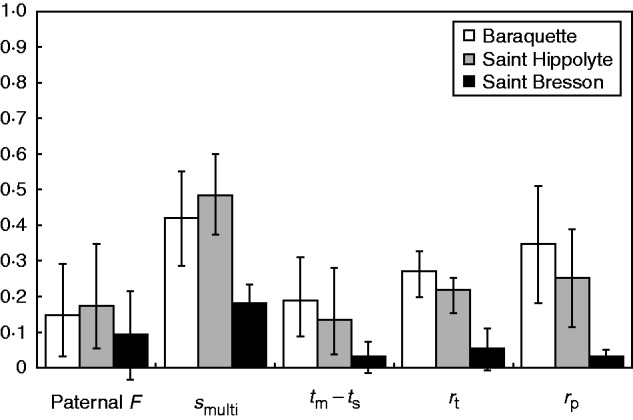
Mating system parameters obtained with MLTR in the BQ (2007), HI (2007) and SB (2011) populations of *Noccaea caerulescens*. Error bars represent the 95 % confidence interval obtained after 1000 bootstraps. *s*_multi_, multilocus estimate of the selfing rate; *t*_m_ – *t*_s_, estimation of biparental inbreeding; *r*_t_, correlation of selfing among families; *r*_p_, correlation of paternity. Analysis was performed on 15 loci for BQ and HI, and 13 loci for SB.

Analysis of the progeny structured data set (Fig. 4) indicated that the paternal inbreeding coefficient *F* ranged between 0·09 (95 % CI 0–0·21) and 0·17 (95 % CI 0·05–0·35), suggesting moderate inbreeding of fathers. Multilocus selfing rate estimation ranged from 0·18 (95 % CI 0·12–0·23) to 0·49 (95 % CI 0·37–0·60). These estimates are within the range of values observed in RMES. From the difference between multilocus and single locus estimates of selfing, we determined that biparental inbreeding varied between 0·03 (95 % CI 0–0·07) in SB and 0·19 (95 % CI 0·12–0·25) in BQ. The correlation of paternity ranged between 0·032 (95 % CI 0·01–0·051) in SB and 0·35 (95 % CI 0·18–0·47) in BQ, which correspond to 31 and three different fathers per mother plant, respectively. The correlation of selfing among individuals of the same family ranged from 0·05 (95 % CI 0–0·11) to 0·27 (95 % CI 0·1–0·43) in the three populations, indicating small to moderate variance in selfing rates among mother plants.

## DISCUSSION

In this study, we characterized the variation in the mating system of *N. caerulescens* in both space and time, in the region of Montpellier, Southern France, by estimating gene flow both between and within populations.

### Gene flow between populations

We found that populations of *N. caerulescens* in Southern France are in general relatively strongly differentiated despite distances of a few kilometres between them, and that metallicolous populations tend to be more differentiated than non-metallicolous populations. The magnitude of genetic differentiation among populations, which is quite typical of plants with a mixed mating system (Loveless and Hamrick, 1984; Hamrick and Godt, 1996), and the lack of isolation by distance point to little gene flow among populations. Such a result was previously found in the same region (Dubois *et al.*, 2003; Jiménez-Ambriz *et al.*, 2007) and at a larger scale (Gonneau, 2014; although isolation by distance was occasionally detected in his data set) in this species, and in some other pseudometallophyte species (Mengoni *et al.*, 2000; Deng *et al.*, 2007; Słomka *et al.*, 2011). Isolation by distance and lower *F*_ST_ values were, however, detected in the self-incompatible *Arabidopsis halleri* at a larger scale (Meyer *et al.*, 2009; Pauwels *et al.*, 2012). Neutral differentiation between ecotype is very small here, suggesting that the ecotype structure does not have much effect on gene flow among populations. This lack or small effect of ecotype on the structure of neutral genetic variation is relatively common in pseudometallophytes (Mengoni *et al.*, 2000; Deng *et al.*, 2007; Bizoux *et al.*, 2008; Słomka *et al.*, 2011); geographical grouping sometimes explains neutral genetic variation better than ecotypic characteristics (Baumbach and Hellwig, 2007; Pauwels *et al.*, 2012). Given that gene flow seems to occur at smaller distances than between polluted and non-polluted sites, we do not expect local adaptation in the metallicolous ecotype here to select for higher selfing rates than in the non-metallicolous ecotype or other types of zygotic barriers, such as described in Antonovics (1968) or Antonovics (2006). Instead, our results suggest that both metallicolous and non-metallicolous populations are distributed as isolated islands in the landscape.

The higher differentiation of metallicolous populations remains to be explained, given their higher effective size and lower inbreeding (see discussion below). The strongly selective environment in contaminated sites, or their ecological isolation, may further restrict gene flow into those populations.

### Gene movement within populations

#### Methodological improvements.

We confirmed that *N. caerulescens* has a mixed mating system using a recent method that is robust to several biases affecting previously used *F*_IS_-derived estimates of mean selfing rate (Dubois *et al.,* 2003). *F*_IS_-derived estimates rely on assumptions such as inbreeding equilibrium, the absence of population sub-division or biparental inbreeding, and are sensitive to genotyping errors, small population sizes and small sample sizes (Jarne and David, 2008; Wang *et al.*, 2012). The method implemented in RMES also assumes inbreeding equilibrium and absence of population sub-division, but population sub-division should bias RMES estimates only if one sub-population is consistently less heterozygous at all loci than the others. We have no reasons to think that it should be the case within our populations. In addition, RMES is robust to the presence of null alleles (David *et al.*, 2007; Jarne and David, 2008) as well as to small sample sizes (Wang *et al.*, 2012), and simulations show that the bias generated by biparental inbreeding is small (Wang *et al.*, 2012). We consequently expect our estimates to be more accurate than are estimates derived from *F*_IS_. Ecotype selfing rate were approx. 25 % smaller in RMES compared with *F*_IS_-based estimations, suggesting that the latter estimations may be upwardly biased by biparental inbreeding, which we could detect using progeny arrays in some populations, and by allelic dropouts, which have been detected in some populations for markers in our data set (Mousset *et al*., 2015).

Multilocus individual heterozygosity is affected by selfing in previous generations (Enjalbert and David, 2000), which could lower our power to detect temporal variation. We found, however, that outcrossing rates are relatively high (>50 %), meaning that estimates of selfing rates are mostly determined by the proportion of individuals that were produced by self-fertilization in the immediately preceding generation, with relatively little influence of earlier generations (Enjalbert and David, 2000). Therefore, we believe that the selfing rate did not vary much in time, as otherwise it would have been detected.

Despite the expectation that selfing rates may vary across reproductive seasons in species with a mixed mating system, due in particular to climatic constraints, and despite observations that they sometimes do (e.g. Cheliak *et al*., 1985; Barrett *et al*., 1993; Eckert *et al*., 2009; Coates *et al*., 2013), such variation is not a general rule (Barrett *et al.*, 1993; Eckert *et al*., 2009). For instance, Eckert *et al*. (2009) found no temporal variation of the mating system in about half of the 30 studies that they reviewed. The stability of the mating system in the presently studied set of populations, however, remains to be tested for a greater number of years and populations.

#### Mating system in *N. caerulescens*.

Mean selfing rates of the ten *N. caerulescens* populations estimated in RMES and MLTR ranged from 0·18 to 0·51, and biparental inbreeding (approximated by the amount of apparent selfing based on the comparison of single locus and multilocus estimates) ranged from zero to 0·19. Selfing rates were similar through years and among populations of the same ecotype, but the metallicolous ecotype had a lower selfing rate than did the non-metallicolous ecotype (MET, 0·25; NONMET, 0·4), as previously found by Dubois *et al.* (2003), despite their less robust methodology. Besnard *et al*. (2009) measured *F*_IS_ for 17 populations of *N. caerulescens* in the Jura Mountains (Switzerland): they also found a mixed mating system (selfing from 0 to 0·90) but did not report any correlation between *F*_IS_ and soil metal concentration. Contrasting selfing rates in metallicolous and non-metallicolous populations with contrasted soil toxicity in other parts of the species range would be necessary to evaluate the generality of the pattern that we have documented regionally.

Differences in selfing rates between ecotypes could stem from differences in plant traits affecting pollinator behaviour and self-fertilization mechanisms such as autonomous selfing, or from purely external factors such as differences in pollinator and plant communities in polluted and non-polluted sites.

Variation in mating system between ecotypes could be due to variation in inbreeding depression. Inbreeding depression is a major factor driving mating system evolution (Lande and Schemske, 1985; Goodwillie *et al.*, 2005; Charlesworth, 2006), and it varies strongly with environmental conditions (Armbruster and Reed, 2005; Fox and Reed, 2011). Selfing rates were measured on adults collected in the field. We would underestimate selfing rates if inbreeding depression were acting on traits affecting survival before sampling (such as seed survival, germination or juvenile survival) and thus eliminating inbred individuals. Different levels of inbreeding depression in metallicolous and non-metallicolous populations could thus, in part, explain the differences in estimated selfing rates between the two ecotypes. If we assume no inbreeding depression in the non-metallicolous ecotype and the same initial selfing rate of 0·4 for both ecotypes, inbreeding depression for survival in the metallicolous ecotype would have to reach 50 % to explain the difference in estimated selfing rates between the ecotypes when sampled at the adult stage. Preliminary estimates of inbreeding depression in *N. caerulescens* suggest that inbreeding depression for survival and its variation between ecotypes is not sufficient to explain entirely such differences in estimated selfing rates (M. Mousset, unpubl. res.). This strengthens our observation that the two ecotypes do differ in their mating system. If inbreeding depression were higher in metallicolous populations, this could, however, have selected for plant traits resulting in lower selfing rates in these populations.

Variation in plant traits may explain differences in mating system between ecotypes. Traits directly modifying the mating system include floral morphology, floral display (Elzinga *et al.*, 2007; Goodwillie *et al.*, 2010; Devaux *et al.*, 2014) or phenology, which all can increase pollinator attraction or, alternatively, enable autonomous selfing. Dubois *et al.*, (2003) showed that metallicolous populations from the Causses and Cévennes region had a higher ratio of pollen to ovules than non-metallicolous populations, and concluded that this observation is consistent with a more allogamous mating system. Jiménez-Ambriz *et al.* (2007) observed a marginally higher number of flowering stalks in the field for a metallicolous population of *N. caerulescens* from the same region as in our study. It could increase attraction to pollinators, thus increasing outcrossing if more pollinators reach the patch and forage by switching between plants. A larger floral display was, however, sometimes found conversely to increase geitonogamous selfing, i.e. selfing among flowers of the same plant (de Jong *et al.*, 1993; Harder and Barrett, 1995; Galloway *et al.*, 2002; Lau *et al.*, 2008; Devaux *et al*., 2014), so the role of this trait in explaining differences in mating system among ecotypes remains to be tested.

Differences in pollination service in the two types of sites could lead to differences in selfing rates. Pollinators determine the outcrossed fraction of seeds, but may also be responsible for an unknown fraction of selfing, through facilitated selfing (pollinator-mediated within-flower selfing) and geitonogamous selfing (pollinator-mediated between-flower selfing). Differences in diversity, abundance, identity, efficiency or behaviour of pollinators can thus affect self-fertilization rates directly. Metal concentrations in the soil or hyperaccumulating plants may directly affect pollinators through unintentional ingestion of trace elements, or indirectly modify pollinator community through modification of plant community. The diversity, demography and abundance of solitary wild bees were found to decrease along two gradients of heavy metal pollutions (Moroń *et al.*, 2012, 2014). These results do not mean we should always expect higher selfing rates in metallicolous populations due to reduced pollination service (Dulya and Mikryukov, 2015). Indeed, some pollinator species are expected to adapt to the edaphic conditions, and the effects on mating system would depend on species identity, abundance and behaviour toward the considered plant species (Meindl and Ashman, 2015). Further studies of plant and pollinator cohorts, as well as their phenologies are needed to characterize pollination accurately in the two ecotypes of *N. caerulescens* and their effects of mating system.

Pollinator behaviour further depends on the spatial distribution of flowering species and thus on factors including density, population fragmentation or marginality. Dubois *et al.* (2003) noted that non-metallicolous populations of *N. caerulescens* seem to have lower densities than metallicolous populations, which could partly explain differences in selfing rates observed between ecotypes. A test of the effect of density on mating system at the fine spatial scale is currently performed in several metallicolous populations of *N. caerulescens* of Southern France.

### Consequences of mating system differences for adaptation

Does the moderate difference in selfing rates observed in metallicolous (25 %) and non-metallicolous (40 %) ecotypes impose different constraints on adaptation and demography of the two ecotypes? Selfing causes non-random gamete sampling and hitchhiking effects, which decreases the effective population size. Hitchhiking effects are however weak for intermediate selfing rates as estimated in both ecotypes (Hartfield and Glémin, 2014). All else being equal, non-metallicolous populations would then suffer from a moderate decrease in effective size of about 10 % compared with metallicolous populations due to their more inbred mating system (Charlesworth, 1993, cited in Glémin and Ronfort, 2013). This moderate decrease of effective size should have little effect on the probability of adaptation proceeding from standing variation in the two ecotypes. Our estimates of effective sizes from temporal variance in gene frequency suggest that non-metallicolous populations indeed have a reduced effective size compared with metallicolous populations. Even though the latter estimates are not very precise, the difference in effective size seems to be larger than that expected only from the difference in selfing rates, suggesting that other factors reduce effective population sizes in non-metallicolous populations, such as demography, patchiness, or variance in reproductive success. In particular, estimates of effective size in non-metallicolous populations may have been affected by the stronger demographic stochasticity in these populations.

The higher inbreeding of non-metallicolous populations may still facilitate adaptation through the fixation of *de novo* mutations due to the increased expression of recessive rare mutations. For instance, metallicolous populations would thus have a 22 % lower probability of fixation of *de novo* beneficial recessive mutations than non-metallicolous populations (for a mutation with selection coefficient s = 0·01 and dominance coefficient h = 0·1, Glémin and Ronfort, 2013). Purging of recessive deleterious mutations in more inbred populations should result in a lower equilibrium frequency of recessive deleterious mutations in non-metallicolous populations (Gillespie, 1998, p. 96), yielding lower inbreeding depression in these populations (e.g. a decrease of 30 % of inbreeding depression at a single locus for h = 0·1; Gillespie, 1998, p.96, cited in Glémin, 2003). However, both data (Winn *et al*., 2011) and theoretical predictions based on multilocus models (e.g. Lande and Porcher,2015; Roze, 2015) suggest that the relationship between inbreeding depression and selfing rate is weak for intermediate selfing rates. The smaller effective population size in non-metallicolous populations may also result in fixation of weakly deleterious recessive mutations, increasing the drift load but further depressing inbreeding depression (Glémin, 2003). In conclusion, all else being equal, non-metallicolous populations may show less inbreeding depression than metallicolous populations, while the consequences of higher selfing rates in non-metallicolous populations on their adaptation are expected to be weak to moderate, depending on the genetic basis for adaptation (new mutations vs. standing variation).

### Conclusions

Our study confirms the mixed mating system of *N. caerulescens* and provides for the first time precise estimates of selfing rates through two different methods in a set of nearby populations in Southern France. We have discussed several alternative hypotheses that may explain the proximate and ultimate causes of ecotypic differences in mating system. These hypotheses are now being tested by measures of inbreeding depression, pollinator communities and behaviour, and plant traits in metallicolous and non-metallicolous ecotypes. Testing whether metallicolous and non-metallicolous ecotypes differ in mating system in other regions of the large geographical range of *N. caerulescens* would allow assessment of the generality of the present pattern and better elucidation its causes.

## SUPPLEMENTARY DATA

Supplementary data are available online at www.aob.oxfordjournals.org and consist of the following. Figure S1: regression of the pairwise *F*_ST_/(1 – *F*_ST_) in both years on the logarithm of the geographic distances between populations of *Noccaea caerulescens*. Figure S2: projection of individuals in the principal components plane of a principal components analysis in *Noccaea caerulescens*. Figure S3: correlation between selfing rates of *Noccaea caerulescens* derived from *F*_IS_ and estimated with the RMES method. Table S1: pairwise *F*_ST_ between populations of *Noccaea caerulescens*.

Supplementary Data
