# Supplementary material for: Lower selfing rates in metallicolous populations than in non-metallicolous populations of the pseudometallophyte Noccaea caerulescens (Brassicaceae) in Southern France
Source: Ann Bot. 2016 Jan 15;117(3):507–19. doi: 10.1093/aob/mcv191 (PMC4765546; doi:10.1093/aob/mcv191)
Supplement: Supplementary Data [file supp_117_3_507__index.html]

Lower selfing rates in metallicolous populations than in non-metallicolous populations of the pseudometallophyte Noccaea caerulescens (Brassicaceae) in Southern France — Lower selfing rates in metallicolous populations than in non-metallicolous populations of the pseudometallophyte Noccaea caerulescens (Brassicaceae) in Southern France — Supplementary Data 

# Lower selfing rates in metallicolous populations than in non-metallicolous populations of the pseudometallophyte *Noccaea caerulescens* (Brassicaceae) in Southern France

## Supplementary Data

files

- Supplementary Data - pdf file
