# Supplementary material for: Lower selfing rates in metallicolous populations than in non-metallicolous populations of the pseudometallophyte Noccaea caerulescens (Brassicaceae) in Southern France
Source: Ann Bot. 2016 Jan 15;117(3):507–19. doi: 10.1093/aob/mcv191 (PMC4765546; doi:10.1093/aob/mcv191)
Supplement: Supplementary Data [file supp_mcv191_mcv191_Supplementary-data.pdf]

SUPPLEMENTARY DATA

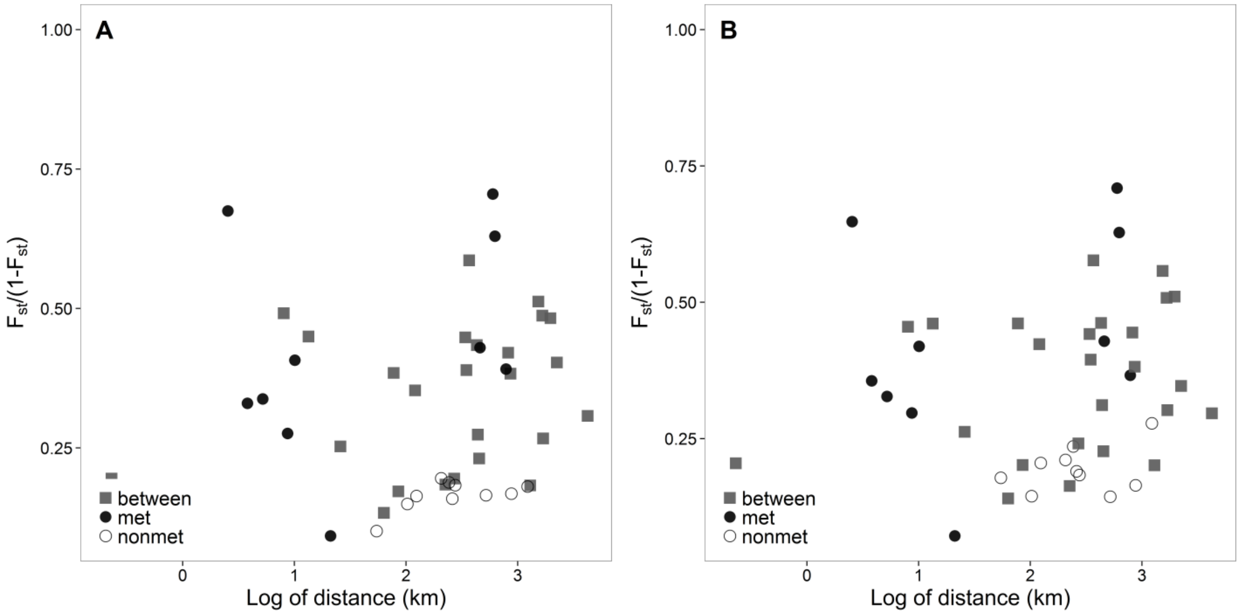

FIG. S1. Regression of the pairwise $F_{st}/(1 - F_{st})$ in both years on the logarithm of the geographic distances between populations of *Noccaea caerulescens*. Light grey, between ecotype; medium grey, among metallicolous populations; dark grey, among nonmetallicolous populations; (A) 2012; (B) 2013. Slopes were not significantly different from zero.

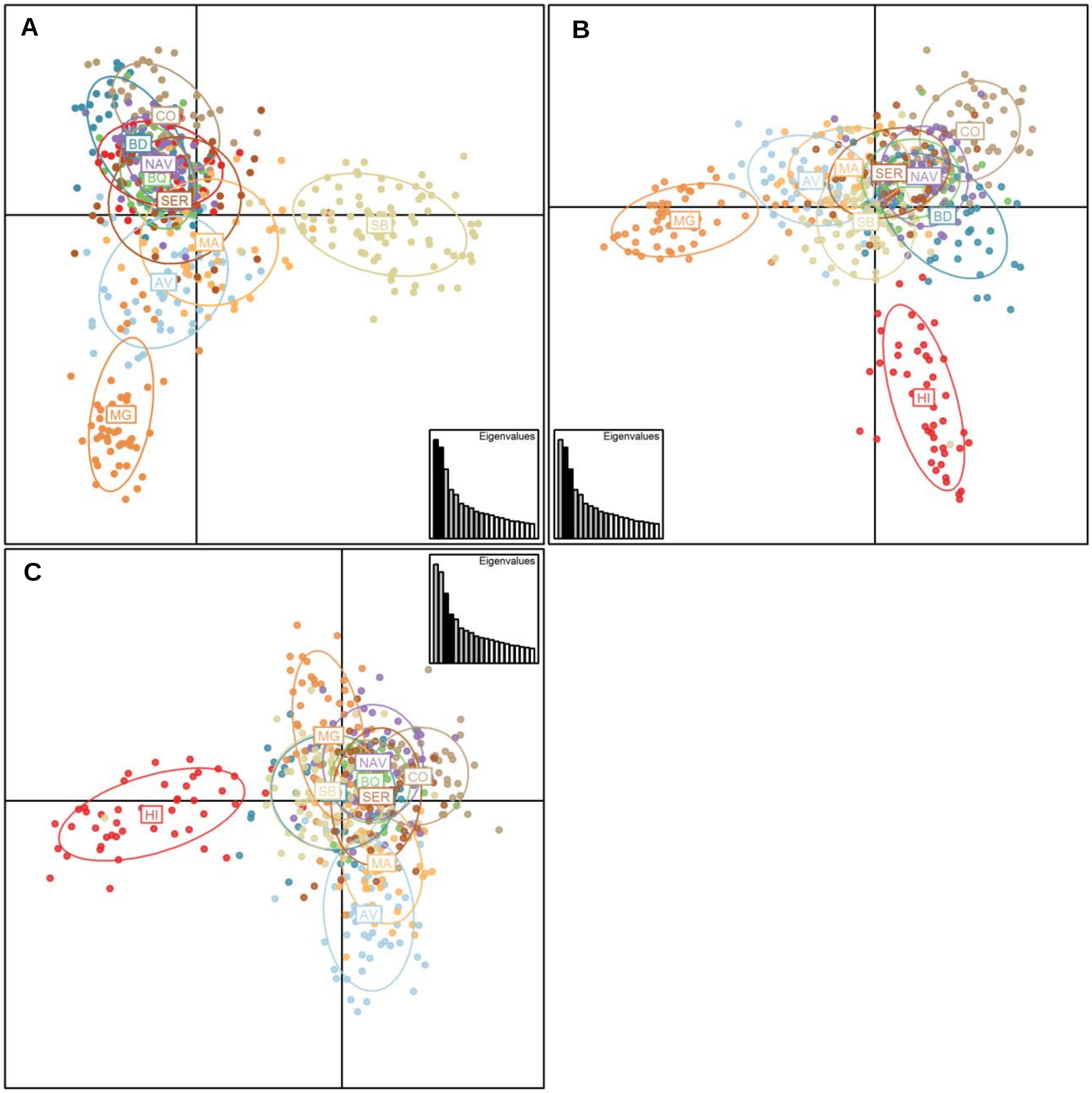

FIG. S2. Projection of individuals in the principal components plane of a principal components analysis in *Noccaea caerulescens*. (A) First and second principal components. (B) Second and third principal components. (C) Third and fourth principal components. Colours represent different populations. Corners: screeplot of eigenvalues associated with each component. Plots from 2013 sampling are not shown as they were extremely similar to those of 2012. AV, Avinières; BD, Saint Baudille; BQ, Baraquette; CO, Coulet; HI, Saint Hippolyte; MA, Malines; MG, Moyen-Âge; Pop, populations ; RT, Route; SE, Sérranne.

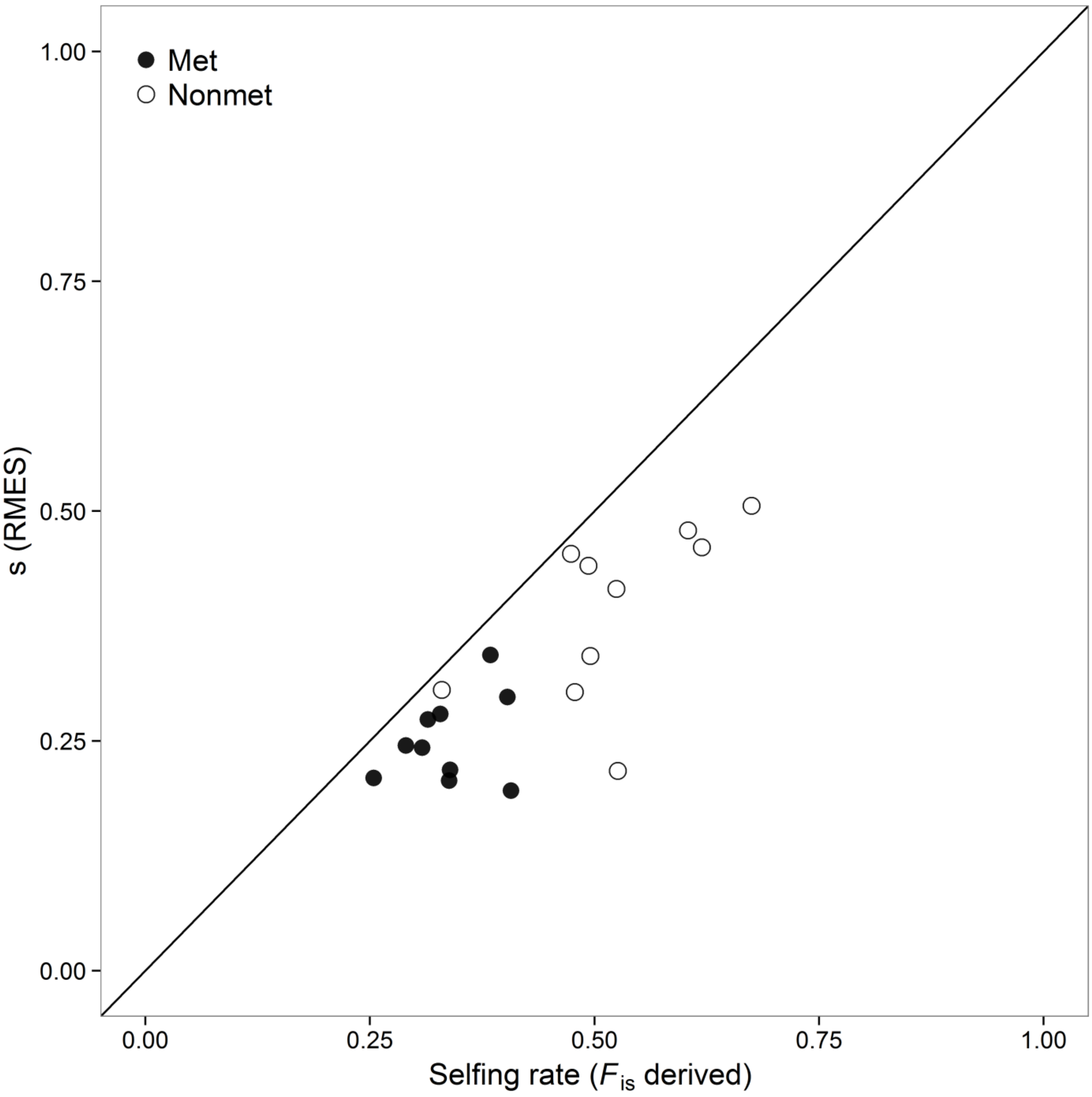

FIG. S3. Correlation between selfing rates of *Noccaea caerulescens* derived from F_{is} and estimated with the RMES method.

TABLE S1. Pairwise F_{st} between populations of *Noccaea caerulescens*. The bottom left half are estimates from 2012 and right top half are estimates from 2013.

		MET					NONMET				
**Ecotype**	**Pop**	**AV**	**HI**	**MA**	**MG**	**SB**	**BD**	**BQ**	**CO**	**RT**	**SE**
MET	**AV**		0.30	0.07	0.26	0.30	0.23	0.21	0.24	0.18	0.17
	**HI**	0.30		0.27	0.42	0.39	0.23	0.31	0.34	0.26	0.28
	**MA**	0.08	0.28		0.25	0.23	0.17	0.17	0.19	0.14	0.12
	**MG**	0.25	0.41	0.25		0.39	0.36	0.31	0.37	0.31	0.32
	**SB**	0.29	0.39	0.22	0.40		0.34	0.32	0.32	0.28	0.30
NONMET	**BD**	0.21	0.24	0.15	0.34	0.33		0.22	0.16	0.13	0.14
	**BQ**	0.20	0.30	0.15	0.33	0.31	0.15		0.19	0.17	0.15
	**CO**	0.22	0.33	0.16	0.37	0.30	0.14	0.16		0.13	0.17
	**RT**	0.19	0.29	0.16	0.31	0.28	0.14	0.16	0.13		0.15
	**SE**	0.15	0.28	0.12	0.28	0.26	0.14	0.09	0.14	0.15	

AV, Avinières; BD, Saint Baudille; BQ, Baraquette; CO, Coulet; HI, Saint Hippolyte; MA, Malines; MET, metallicolous populations ; NONMET, nonmetallicolous populations ; MG, Moyen-Âge; Pop, populations ; RT, Route; SE, Sérranne.
